# Bridging cognitive, phenomenological and psychodynamic approaches to eating disorders

**DOI:** 10.1007/s40519-022-01379-6

**Published:** 2022-02-18

**Authors:** Giovanni Castellini, Emanuele Cassioli, Eleonora Rossi, Milena Mancini, Valdo Ricca, Giovanni Stanghellini

**Affiliations:** 1 Psychiatry Unit, Department of Health Sciences, University of Florence, Largo Brambilla 3, 50134 Florence, Italy; 2 Department of Psychological Sciences, Health, Territory, G. d’Annunzio University of Chieti and Pescara, Chieti, Italy; 3 Centro de Estudios de Fenomenología y Psiquiatría, Diego Portales’ University, Santiago, Chile

**Keywords:** Eating disorders, Phenomenology, Embodiment, Cognitive-behavioural model

## Abstract

Cognitive, psychodynamic, and phenomenological scholars converged their attention on abnormal bodily phenomena as the core psychopathological feature of eating disorders (EDs). While cognitive approaches focus their attention on a need for “objective” (i.e., observable, measurable) variables (including behaviours and distorted cognitions), the phenomenological exploration typically targets descriptions of persons’ lived experience. According to a new emerging phenomenological perspective, the classic behavioural and cognitive symptoms of EDs should be considered as epiphenomena of a deeper core represented by a disorder of the embodiment. The cognitive–behavioural model is the most studied and, up till now, clinically efficacious treatment for EDs. However, as any coherent and scientifically grounded model, it presents some limitations in its application. Numerous patients report a chronic course, do not respond to treatment and develop a personality structure based on pathological eating behaviours, since “being anorexic” becomes a new identity for the person. Furthermore, the etiopathogenetic trajectory of EDs influences the treatment response: for example, patients reporting childhood abuse or maltreatment respond differently to cognitive-behavioural therapy. To obtain a deeper comprehension of these disorders, it seems important to shift attention from abnormal eating behaviours to more complex and subtle psycho(patho)logical features, especially experiential ones. This characterisation represents the unavoidable premise for the identification of new therapeutic targets and consequently for an improvement of the outcome of these severe disorders. Thus, the present review aims to provide an integrated view of cognitive, psychodynamic, and phenomenological perspectives on EDs, suggesting new therapeutic targets and intervention strategies based on this integrated model. Level of Evidence: Level V.

**Level of evidence** Level V: Opinions of authorities, based on descriptive studies, narrative reviews, clinical experience, or reports of expert committees.

## How to define an Eating Disorder

What is an Eating Disorder (ED)? The issue of the definition of EDs is relevant for clinical practice, to identify targets and levels of treatment, for epidemiology, to establish the burden of these diseases, as well as for basic research, to investigate the etiopathogenesis of these disorders. However, the answer to this question may not be that simple, as EDs can be described according to different perspectives, namely biomedical, behavioral, nosographic and experiential. Thus, the definition of EDs must take this complexity into account.

The Diagnostic and Statistical Manual of Mental Disorders (DSM-5) defined the so-called “Feeding and EDs” as “persistent disturbances of eating or eating-related behaviours that result in the altered consumption or absorption of food and that significantly impair physical health or psychosocial functioning” [1]. This level of definition is based on a behavioural characterisation. Accordingly, clusters of behaviours split out distinct diagnostic categories, namely Pica, Rumination disorder, Avoidant/restrictive food intake disorder, Anorexia Nervosa (AN), Bulimia Nervosa (BN), and Binge-eating disorder (BED). From an epidemiological point of view, the new system for ED classification proposed by the DSM-5 represented a step forward, reducing the number of “ED not otherwise specified” in favour of an increase of the number of patients affected by full-blown diagnoses [2]. However, a large proportion of subjects reporting problems with their eating behaviours continues not to be classified into the main diagnoses [3]. However, the new diagnostic criteria are still based on behavioral features, and it may not be an adequate answer to relevant clinical issues related to prognosis, course, and outcome, as well as to risk factors and early manifestations of these disorders.

More than ten years ago, Fairburn developed a trans-diagnostic psychopathological model for the characterisation and treatment of EDs [4]. According to this model, patients with AN, BN and BED share a common psychopathological core based on pervasive concerns about body shape and weight that influences their eating behaviours. From a clinical and epidemiological perspective, the *galaxy* of all the conditions potentially associated with these core features appears much wider than the one provided by the DSM-5. Indeed, aggregations based on pathological eating behaviours include several phenomenal variants, such as night eating syndrome, purging disorder, atypical anorexia nervosa, non-fat phobic AN, non-purging disorder BN [1]. The whole representation of the ED picture is even more complicated if we consider subclinical disorders. These conditions are estimated to reach a lifetime prevalence of more than 13% in western countries [5], and they resulted particularly represented among high-risk populations, such as ballet dancers, gym attenders or orthorexic subjects [6, 7].

Furthermore, outside the boundaries of this *galaxy* other conditions share psychopathological dimensions commonly associated with EDs. For example, obese subjects and patients with mood disorders often report the tendency to eat in response to different emotions (the so-called *emotional eating* phenomenon) [8]. Moreover, body image disturbances can be found in patients with body dysmorphic disorder [1], depression [9], social anxiety [10] or gender dysphoria [11]. In this latter case, persons sometimes report severe dietary restrictions to change their body appearance [12]. Some authors suggest using the concept of *comorbidity* to explain the existence of psychopathological features shared by individuals with different diagnoses [13, 14]*.* Is this because of the lack of clear boundaries between the disorders? An alternative explanation could be that specificity and boundaries between disorders are not lacking, rather they depend on the level of assessment. The behavioural level is certainly not adequate to separate disorders and to identify their specificity.

## Issues with diagnoses based on behavioural criteria: clinical cases

The use of categorical diagnoses based on behavioural criteria might represent a critical issue in the clinical practice of EDs, as we will show in the following case studies.

Case 1. An 18-year-old girl reporting concerns about her weight and body shape for much of her life. When she was sixteen years old, before a dancing competition, she started dieting, with very rigid diet rules and “danger” or “forbidden” foods. After a while, parents described a severe social withdrawal, with heightened obsessive preoccupations and a progressive marginalisation of other than food control areas of life. The patient reported a dramatic weight loss, reaching in a few months a body mass index (BMI) of 15.6, with amenorrhea.

Case 2. A 22-year-old woman who, after the end of the relationship with her boyfriend, reported being extremely unsatisfied with herself and about her body. For a while, she progressively adopted more and more rigid dietary rules, but suddenly binge-eating episodes appeared, with an uncontrolled assumption of large amounts of cookies, candy, ice creams, cakes and sandwiches with cheese and ham. Then, to manage the feelings of guilt and shame induced by the break of her dietary rules, she started to force herself to throw up after every binge-eating episode. This cycle repeated itself once every week and continued for several months.

Case 3. A 20-year-old woman with a history of Anorexia Nervosa. She attended an outpatient program of nutritional rehabilitation in a private clinic for six months. At the end of this treatment period, she reported regularly eating 1600–1800 kcal a day. The BMI was considered stable around 20, and she recovered regular menses. Even though she was considered recovered according to the DSM-5 criteria, she reported being still extremely unsatisfied about her body, with severe body shape avoidance (she was unable to touch or to look at any part of her body). She still showed over-control on her diet.

Case 4. An 8-year-old child. She was an overweight child since she could remember. She reported a history of neglect by her parents. To manage sadness or other bad emotions, she spent the afternoon watching movies and eating all kinds of available food, such as pizza, potato chips, gallons of ice cream.

The first two cases are prototypical examples of Anorexia Nervosa restricting type (case 1) and Bulimia Nervosa (case 2). The third one is a girl who falls into what Keski-Rahkonen defined as a pseudo-recovery state [15]: a condition in which a person is considered recovered from a behavioural point of view, but who is still “walking the walk” and internally “talking” the same eating disordered talk. The fourth case is not properly an eating disorder, but a person at high risk for developing it.

Even though they were described as distinct clinical cases, these were not different subjects with different diagnoses, but *four cross-sectional representations of distinct stages of a unique disorder referred to the same real person*. Like an underground river, the ED psychopathology came to light in different forms over time, beginning as AN, momentarily disappearing and then suddenly re-emerging as BN. Life events and environmental factors moderated this tortuous course: a neglect condition during childhood, pressure for thinness during adolescence, an event of loss before the onset of BN.

Three main information can be derived from this clinical case: DSM-5 diagnoses are state-dependent conditions (diagnostic instability), they are not associated with specific outcomes (no prognostic value), and they are not associated with specific etiopathogenesis (no etiological value).

Considering that DSM-5 diagnoses tend to be unstable and to change across time, a person can be diagnosed for AN and later develop BN. This phenomenon is called diagnostic crossover, and it is the consequence of the fact that behaviours change over time [2, 16, 17]. Diagnostic instability is quite strange in the field of medicine, where in general a disorder can evolve into a more severe condition, but not into a different disorder, e.g., cancer can develop metastasis, but a non-Hodgkin lymphoma cannot become a Hodgkin lymphoma. Movement from one diagnosis to another is a consequence of dividing patients on the basis of BMI and behavioural characteristics (underweight patients, patients who binge and vomit and patients who binge without vomiting), and it really does not provide complete information regarding the recovery process of a patient.

Indeed, several studies demonstrated that weight, shape [18] and eating concerns [19, 20] persisted altered in those patients who were considered only biologically and behaviourally but not cognitively remitted [21].

In general, this is a problem in psychiatry. Diagnoses should provide information regarding prognosis and treatment targets [22]. However, the actual diagnostic system for EDs uses clusters of phenotypes that are neither related to etiological factors or longitudinal courses and outcomes nor to treatment response. In other areas of medicine, things are different. For example, considering lung cancer, all the available classifications provide information regarding aetiology (e.g., smokers vs non-smokers), possible treatment strategies (e.g., cancer subtypes more sensitive to chemotherapy) and prognosis (life expectancy is often associated with different histological types).

Finally, considering the multifactorial etiopathogenesis of EDs, to date, there is very scant evidence of a clear association of genetic polymorphism with specific DSM-5 diagnoses and even with the ED category itself, as compared with other psychiatric disorders [23–25]. The same observation can be made for neurobiological alterations such as HPA dysfunctions [26] or for well-known risk factors such as childhood abuse or neglect, which are often reported in EDs [27, 28] but also in major depression or post-traumatic stress disorder [29, 30].

## A phenomenological assessment of EDs psychopathology

While cognitive approaches focus their attention on the “objective” (i.e., observable, measurable, quantifiable) core features of EDs, the phenomenological exploration typically targets self-descriptions of persons’ lived experience. A phenomenological assessment of the patients’ experiences results in a rich and detailed collection of patients’ self-descriptions, which reveal fundamental changes in the structures of subjectivity and can be captured using specific assessment tools [31–33]. A comprehensive phenomenological assessment of EDs should focus on the subjective experience of one’s own body (lived body, i.e. the way one almost implicitly feels one’s own body in the first-person perspective) and of one’s body in space (lived space, i.e. space as experienced not as an objective coordinate system but as the practical, meaningful space of everyday life), as well as on the subjective experience of time (lived time, the way we experience time in a subjective way rather than the way we observe it on the clock in an objective way) associated with abnormal eating-related behaviours (e.g. starvation, binging, body weight and shape control). It should also focus on the relations between abnormal body experiences and representations and the construction of personal identity [34]. Yet, clinical phenomenology is by no means restricted to the mere description of abnormal phenomena. As an approach that investigates the patients’ subjective experience, indeed their everyday existence in a comprehensive way can go one step further and provide the basis for an extended understanding of their way of being in the world that is useful for personalised clinical practice [35]. Thanks to this approach, clinicians might move a step forward to understand the personal meaning of EDs symptoms, envisioned from the patient’s perspective [36].

Given the deep interpenetration of embodiment and spatiality, for example, regarding their body in space, some patients report that they “cannot step through the door” or that they “take a lot of space in a room”. The fear of a metamorphosis of their body in space is compensated by repetitive, time-consuming, and compulsive rituals. Examples of these ritualistic behaviours include lifting weights, excessive mirror checking or avoidance, comparing one’s appearance with that of others, seeking reassurance about weight fluctuation, skin picking, camouflaging the changes of lived body in space (e.g., with frequent adjustments of makeup, body posture, position, clothing, etc.).

Also, the temporal continuity of bodily experiences is altered in patients with EDs. Temporal continuity has been reported as subjectively experienced as uncontrolled or discontinuous. For example, patients report the feeling that their body can change continuously [37]. Thus, the inner experience of time is associated with a subjective perception of control and/or loss of control on one’s body, which motivates hyper-control of shape, weight or feeding: “One morning I feel my thighs fit perfectly in my pants, another morning instead they have become huge”. The comprehension of the dynamic interplay of body experience and contextual events might be an essential perspective for the interpretation of ED psychopathology. Patients with EDs often report a subjective experience of body fluidity in everyday life. As the temporal continuity of body experience is altered in patients with EDs, interpersonal relationships are also affected since there cannot be a way for being with the others in simultaneity or in a flowing succession of temporality [38].

A good example of integration of cognitive and phenomenological perspectives on EDs might be represented by a different use of diaries for food and eating habits, which are often adopted as a self-monitoring strategy of restraint and binge-eating episodes in CBT. The qualitative analyses of food diaries may provide a deeper description of patients’ subjective experience, allowing a more precise characterisation of triggers of body uneasiness and of pathological eating behaviours [39].

For example, instead of categorising binge-eating in terms of quantity of food (objective vs subjective binge-eating) [40, 41], it would be clinically meaningful to focus on the patient’s experience of lack of control (including threatening changes in bodily feelings, fluctuations in space and time experience, emotional antecedents and consequences of the binge episode).

The dimension of control crosses the boundaries of the cognitive area. Indeed, it is shared with the psychodynamic literature, which represented patients with AN as persons reporting a pervasive sense of uncertainty and instability in their life [42]. Williams et al. [43] showed that individuals with any ED perceive a low degree of internal control and high external control exerted by family and society.

## The body is the battleground of eating disorders: expanding the concept of body image

Cognitive, psychodynamic, and phenomenological scholars converged their attention on abnormal bodily phenomena as the core psychopathological feature of EDs [36]. Overall, clinical research referred to the generic term of *body image disturbance* as a cognitive-affective attitude toward one’s own body that embraces various concepts related to negative body image, such as body dissatisfaction, avoidance, or, on the contrary, compulsive control of one’s own body, detachment, and estrangement feelings towards it and worries about particular body parts, shapes or functions [44]. All these psychopathological dimensions seem to pertain to a core feature preceding the onset of behavioural symptoms [45], often persisting after treatments [46], and being associated with a worse prognosis [2, 47] and with a higher likelihood of relapse after remission [48].

A severe bias to the interpretation of results arising from clinical studies is the heterogeneity of measures related to body image and the unspecificity of the so-called “body uneasiness” in EDs. For example, the *Body Uneasiness Test* has been administered to persons with gender dysphoria [11], who were found to score at similar levels of persons with AN, BN or BED. Uneasiness towards one’s own body has been described as a dimension lying as a continuum in the general population without any specific threshold for clinical conditions [13]. Moreover, it was associated not just with EDs but also with obesity [49], social anxiety [1], schizophrenia [38, 50], manic-depressive disorders [51], and body dysmorphic disorder [52].

Scholars proposed that *body image distortion*—defined as “a disturbance in the way in which one’s body weight or shape is experienced”—might represent a specific dimension to distinguish EDs from other disorders [53]. However, neither this seems to be a specific construct if taken as a monolithic entity, considering its multidimensional structure that includes components regarding beliefs and emotions (concerns and feelings about the body), perception (estimation of body size) and behaviours related to body perception.

What is distorted about the body in EDs? If we want to establish a correlation between abnormal phenomena and their neurobiological substrate, the former must be rigorously defined. This has not been the case with translational research in EDs, and as a consequence of this, neurobiological research got lost in the heterogeneity of bodily abnormalities involved (and related constructs like e.g. body image, body uneasiness, etc.) and in the plethora of different brain areas supposedly implicated in their pathogenesis (including dorsolateral, prefrontal, supplementary motor, insular, inferior parietal, fusiform, occipito-temporal, and cingulate regions) [54].

Neurocognitive research attempted to provide a solution proposing a further construct called *body schema* [55, 56], defined as “a system of sensory–motor capacities that functions without awareness or the necessity of perceptual monitoring” [57]. Body schema is an implicit representation of our body which is the result of comparisons and integration of past sensory experiences (postural, tactile, visual, kinaesthetic and vestibular) with current sensations at the cortical level [38, 58]. From the cognitive perspective, it has been extensively noticed that changes in body schema affect spatial perception and perception of objects. Exercise, dance, and other practices that affect motility and postural schema have an effect on the emotive evaluation of one’s own body schema [59, 60]. On the basis of these observations, Gallagher [57] argued that performances of the body schema might place constraints on intentional consciousness and suggested that changes in various aspects of body schema affect the way subjects perceive their own bodies, that is, changes in body schema lead to changes in body images.

However, as previously reported, it is clear that the disturbance of body image in EDs cannot simply be ascertained from a somatic-sensory alteration or a failure to integrate somatic sensations at different levels. Since 1893, Bonnier [61] rejected the idea that the conceptualization of one’s body is simply the sum of somatic sensations arising from it.

**Fig. 1:**
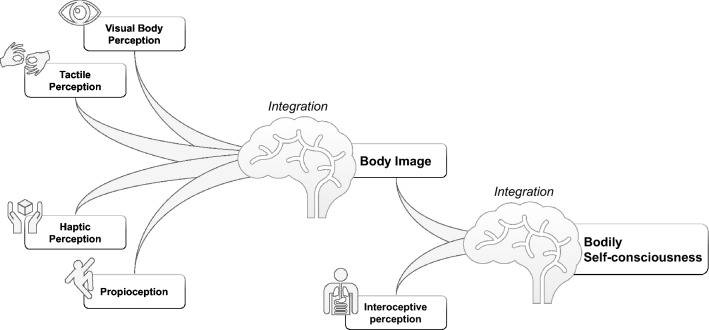
Components of body image and bodily self-consciousness

Thus, problems associated with body experience should not be considered as affecting merely a topography of the body, rather they might involve a complex pattern of encoding and integration anomalies of a wide range of multisensory-somatosensory, visual, auditory, vestibular, visceral, and motor signals [62] (Fig. 1). Clinicians and researchers traditionally tend to explore a mere visual distortion of the body of their patients, ignoring tactile and haptic perception and proprioception. However, empirical evidence supports a more complex view of *body uneasiness* in EDs. For example, the degree of body dissatisfaction was found to be associated with the severity of tactile perception inaccuracies in patients with AN [63–65]. On the other hand, haptic reproduction abilities have been found to be poorer in patients with AN as compared to controls both before and after weight gain [66]. Regarding proprioception—referred to the sense of limb and body position in space [67]—impaired spatial orientation constancy was found in patients with AN compared to controls [67].

Clinicians listening to their patients’ narratives regarding their anomalous body experiences (e.g. feelings of changes in their body after a meal or in relation to dysphoric mood) should be aware that they are talking about something more complex than a *visual representation* of the body [58]. The integration of body visual, tactile, and haptic perception with proprioception which structures the so-called *body image* is not sufficient to structure our *bodily self-consciousness* as a whole [62]*.* These must be integrated with *interoception* to reach this further step. Interoception includes a range of sensations, such as heartbeat, temperature, intestinal tension, hunger, or pain. These sensations are often concomitant and interconnected with emotional responses. It is well known that patients with EDs often report poor interoceptive awareness, with difficulties to discriminate sensations related to hunger and satiety [68, 69]. It can be hypothesised that patients having difficulties in the discrimination of visceral sensations with respect to hunger and satiety should also be less able to perceive bodily signals in general, including emotional arousal [70]. According to Damasio's somatic marker hypothesis, interoception is regarded as an essential component of subjective emotional perception. Perceiving changes in the bodily state (autonomic bodily signals) is the basis of our emotional experiences [71, 72]. Indeed, we feel emotions because we perceive our bodily reactions [73]. Persons who perceive bodily signals with a high degree of sensitivity experience emotions more intensely, while reduced interoceptive awareness is associated with an affected experience of emotions [71, 72]. For example, the insula is a common neurobiological underpinning for both emotional states and interoception, since it is involved in several different emotional reactions, such as happiness, hanger, fear, disgust, as well as in the perception of pain, itch, sexual arousal, feelings of coolness or heat, sensations related to respiration or exercise [74, 75].

This broader concept of interoception might represent the bridge between the reduced sensitivity and awareness to body signals in patients with EDs and their scarce competence in emotion recognition and modulation. Authors demonstrated this intercorrelation from several points of view. In particular, ED specific psychopathology was found to be associated with different measures of emotional non-competence, including emotion dysregulation, alexithymia and impulsivity [76–78]. Patients such as the one mentioned above often fail to differentiate their body sensations from their emotions, reporting statements such as “I used salty and full-bodied foods in the moments of anxiety, while the sweet, warm, soft or liquid prevail in conditions of sadness”.

Emotion recognition is an essential step in the process of pre-reflexive self-constitution and reflexive self-definition, and the primary experience of the body is constitutive of self-consciousness [72, 79]. This complex and dynamic process involving self-constitution and self-definition moves its first steps in childhood through different processes, such as self-identification, self-location, self-perspective, self-demarcation, self-agency [62]. Adolescence—the crucial age for developing an ED—represents the stage in which the self becomes abstracted from the body and is intellectualised as the self-conscious mind [80].

Thus, we can conclude that the problem of persons with EDs is not just body uneasiness and that what is distorted is not just a visual image of one’s body. Clinical and laboratory evidence demonstrates that persons with EDs show an altered bodily self-consciousness, which is probably already present years and years before the onset of the disorder. Anomalies of bodily self-consciousness convey a sense of extraneousness toward one's own emotions, and this is associated with problems in self-definition, which is dysfunctionally managed in some vulnerable persons through the development of ED symptoms.

## A new integrated model for eating disorder psychopathology

Scientific research is now facing an impasse in its attempt to identify, objectify, and measure abnormal bodily phenomena. A new perspective could be helpful to overcome these difficulties. According to a recent hypothesis [81–83], the way of living one's own body for persons with EDs is not *distorted*, rather it is *disproportionate*, with a prevalence of optical representations and a detriment of the coenaesthetic experience of the body [62, 81, 82].

### Disorders of the subject-body in persons with EDs

Traditionally, phenomenology distinguishes between two forms of embodiment—the lived body (*Leib*) and physical body (*Koerper*), or subject-body and object-body [84]. ‘Subject-body’ represents the coenaesthetic apprehension of one’s own body—the primitive experience of oneself as a spatiotemporal embodied agent in the world and the basic form of self-awareness. The ‘object-body’ is the body thematically investigated as an entity existing in the outside world, as for example by the natural sciences like anatomy and physiology. Whereas the experience of my subject-body is a direct, unmediated apprehension of it in the first-person perspective, neither a representation of it mediated by reflection (as the case with ‘body image’) nor the perception of my body as an external entity separated from myself; I experience my object-body in the third-person perspective. *Sight* is the principal sense modality through which I perceive my body as an object-body, whereas the modality by which I apprehend my body as subject-body is called *coenaesthesia*.

First and foremost, the way we experience our body is the outcome of the dialectics between coenaesthesia and sight. Bodily experience is a combination of the way we feel ourselves from a first-person perspective and the way we see ourselves from a third-person perspective. This is a way to transcend our private and idiosyncratic apprehension of ourselves.

It is a common observation that patients with AN appear to be surprised when clinicians provide *objective* measures of their body, reporting for instance renal and hepatic flaws, hypoglycaemia, hypokalaemia, pericardial flare, or other dangerous alterations in their object-body. They are often astonished or even shocked when their body is biomedically objectualised and measured. “Only if I see my body turned into a series of numbers, for instance after a blood test, I realise that I am sick”, says a patient. When the doctor has pointed out and shown them their severe underweight, severe dehydration, dental problems, oedema, and eventually heart complications or blood alterations, ED patients become aware of their body in a new and disturbing way as it now turns into a physical object.

Phenomenological analyses suggest the relevance of the distress related to the subject-body in ED patients and concentrate on *abnormal bodily experiences*, which are usually at the margins of assessment instruments mainly focused on anomalies of body image and schema.

### Unbalance of subject- and object-body in person with EDs

Under normal conditions, there is a dialectic equilibrium and balance between the experience of one’s body from a first- and a third-person perspective, a proportion between the subject- and the object-body. In this perspective, the imbalance between the external and internal apprehension of the body related to ED symptomatology described by Eshkevari et al. could be re-conceptualised as a disorder of the coenaesthetic apprehension of the body [85]. Given that bodily self-consciousness is a core component for encoding all emotional feelings [72, 79], this may explain why people with EDs have difficulties in the identification and expression of their emotions, with consequent disruption of their sense of identity.

There is an interesting convergence of this phenomenological view with neuroscience, in particular with the conceptualisation of the so-called egocentric and allocentric way to access one’s own body. The egocentric frame (body as a reference of first-person experience) is perceptive/experiential since it has its primary source on somato-perceptions, as first-person experience of oneself, while the allocentric frame (body as an object in the physical world) is representational since it has its primary contents in somato-representations—abstract knowledge, beliefs, and attitudes related to the body as an object of third-person experience (e.g. look oneself in the mirror or visual images memories). In persons with EDs, a predominance of the allocentric frame seems to be present, and the distorted experience of the body is a consequence of the impairment of the process of integration between the egocentric experience of the body and the allocentric representation of it.

### The lived-body-for-others: the other’s gaze as an optical prosthesis of the subject-body

It has been also hypothesised [34] that in persons with EDs, next to an unbalance between the subject- and the object-body, there is a predominance of a third dimension of embodiment, namely the lived-body-for-others. This is a concept proposed by philosopher Sartre [86], in addition to the abovementioned subject-body/object-body distinction. Sartre emphasised that one can apprehend one's own body as one's own body when it is *looked at by another person*. This further profile of self-experience happens when people realise that their own body can be observed from the perspective of another person, and therefore it can be an object for others. The lived body is no longer a direct, first-personal experiential evidence, but it is an entity that exists as viewed from an external perspective [34].

One feels oneself and becomes aware of one’s body, thanks to the experience of being seen by another person. In this light, the specific alteration of embodiment detected in patients with EDs can be seen as the experience of one’s own body experienced first and foremost as an object being looked at by another, rather than coenaesthetically apprehended from a first-person perspective [34]. In this perspective, thinness and related strategies, such as starvation, vomiting or purging, are used to shape one’s bodily appearance in an attempt to (re)gain a sense of bodily self as an object evaluated from the perspective of the other’s gaze. An imbalance between the external and internal perception of one’s own body is related to ED symptomatology severity and also persists after weight recovery [87].

**Fig. 2:**
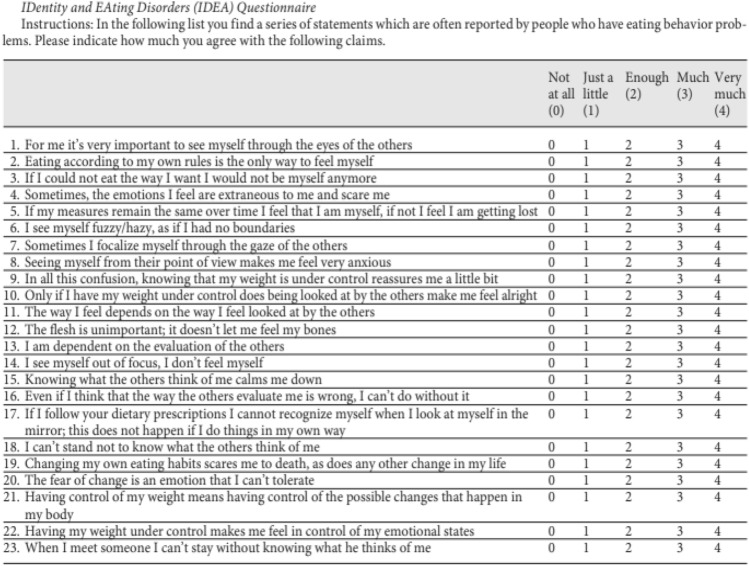
IDentity and EAting Disorders (IDEA) questionnaire

Based on this theoretical background, our group [34] validated a questionnaire named IDentity and EAting disorders (IDEA), assessing abnormalities in embodiment and personal identity (Fig. 2). The questionnaire was developed based on the following conceptual areas: feeling oneself through the gaze of the other, defining oneself through the evaluation of the other, feeling oneself through objective measures, feeling extraneous from one’s own body, feeling oneself through starvation, defining one’s identity through one’s own body, feeling oneself through physical activity and fatigue. Theoretically, the questionnaire assumed that most pathological eating behaviours and features are a consequence of the severity of abnormal bodily experiences and identity disorders. The authors demonstrated that the questionnaire was able to identify important psychopathological phenomena that are closely related to the specific anomalies of patients with EDs measured with commonly adopted psychometric instruments for EDs psychopathology such as the Eating Disorder Examination Questionnaire [34] and Eating Disorder Inventory [88, 89]. These results were confirmed for patients reporting AN, BN and BED [34]. In a second step, the questionnaire was also tested beyond the boundaries of full-blown diagnoses to evaluate its utility in detecting a core EDs psychopathology also outside the clinical conditions.

Among a population of university students, IDEA was able to identify vulnerability in subjects without full-blown EDs but with abnormal eating patterns [90], thus characterising candidate experiential intermediate phenotypes which express a gradient of vulnerability from healthy to clinical persons with EDs. The questionnaire was applied to morbidly obese patients, individuating those subjects with more vulnerability to pathological eating behaviours [91].

### Subject-body and personal identity: EDs as surrogate identity

The unbalance between apprehending one’s body coenaesthetically (from within) and as an object to be seen (from without) was named *optical-coenaesthetic disproportion* [81–83]. From the angle of the optical-coenaesthetic disproportion hypothesis [62, 82], behavioural anomalies in patients with EDs, as well as the excessive importance attributed to body shape and weight, should be considered as secondary epiphenomena of this deeper psychopathological core represented by the disorder of the coenaesthetic apprehension of one’s body. According to this hypothesis, the disturbance of the experience of one’s own subject-body is interconnected with an impairment in the process of shaping personal identity. As previously reported, the interplay between embodiment and self-identity is supported by neuroscience evidence [62, 92]. Indeed, the incoming multisensory bodily signals shape bodily self-consciousness, which has been indicated as the implicit, pre-reflexive background of *selfhood* [62, 92]. The conceptualisation of ED core psychopathology as a problem of self-identity can be derived from different perspectives. Overall, both cognitive and psychodynamic theories consider that ED symptoms are maintained by a dysfunctional system for evaluating self-worth. From a psychodynamic perspective, Bruch suggested that the dissatisfaction with body image that characterises persons with EDs reflects a maladaptive “search for selfhood and a self-respecting identity” [93]. According to Fairburn (cognitive-behavioural perspective), whereas most people evaluate themselves because of their perceived performance in a variety of domains of life (e.g., the quality of their relationships, work, parenting, sporting ability, etc.), people with EDs judge themselves largely, or even exclusively, in terms of their eating habits, shape or weight and their ability to control them [94].

According to the phenomenological perspective, the experience of not feeling one’s own body and emotions often reported by ED patients [34, 95] entails the impairment of the pre-reflexive constitution and reflexive construction of one’s sense of identity, which is no longer a solid psychic structure that persists beyond the flow of time and circumstances. Therefore, abnormal eating behaviours flourish as a way to shape one’s own identity as a “concretised metaphor”, establishing an equivalence between a psychic reality (identity) and a physical one (body shape) [96–99].

Stanghellini et al. [34] moved a step forward from this position: not just eating behaviours represent a kind of coping strategy to regain a sense of identity, rather the ED condition itself becomes a way to define oneself [100]. Clinicians are aware that patients often report not to suffer from AN, rather they “are anorectic”, or they “need to be seen as anorectics or bulimics”. Anorexia thus becomes for persons who cannot rely on their bodily experiences to build their personal identity a *surrogate identity. This is supposedly a mechanism of maintenance of anorexic symptoms and a reason for chronicity.*

The basic concern of AN persons is feeling shapeless in a concrete (i.e., fat, loose-fitting) and metaphorical sense (i.e., without a definite personal identity). Feeling amorphous in a physical sense and selfless in an existential one are vulnerability traits in persons with AN. AN is a special kind of disembodied existence. Embodiment is the condition of possibility for the experience of appetite, vital energy, a point of orientation for one’s actions and choices and the correlated pattern of meanings that make for a coherent and significant Self in the world. It is what grounds human motivation and organises our experiential world in accordance with needs and wishes, thereby giving objects their “affordances”. In the absence of this vital self-affection and the lines of orientation it establishes, the structured nature of Self and world will be altered or even dissolved.

Specific abnormalities in embodiment, namely experiencing one’s own body first and foremost as an object being looked at by another (rather than coenaesthetically and from a first-person perspective), and of personal identity, namely defining one’s own self largely in terms of the way one feels looked at by the others and through one’s ability to control one’s shape and weight (rather than through other kinds of performance), are supposedly secondary phenomena of anorexia and represent a reaction to cope with this vulnerability.

In the value system of anorexia, persons being fat and eating have an explicit moral value, not simply an aesthetic one. Fatness is seen as indicative of laziness, lack of self-care, or lack of self-control, and therefore contemptible and disgusting, and likely to lead to unpopularity with peers as well as moral contempt. Starvation is seen as a salvation practice since it can help regain a sense of self-worth and authenticity [101]. These values are themselves rooted in and sustained by the cultural value of ocularcentrism, which puts the sense of sight front stage and downplays the role of coenaesthesia and of embodiment in general. Next to these personal and explicit values, ocularcentrism is an implicit value reflecting contemporary societal and cultural ones. The way persons with anorexia experience themselves and the world reflects an unbalance between the optic and the coenaesthetic modalities with a prevalence of the first over the second—thus it reflects the value implicit in ocularcentrism [100]. Persons with AN may be unaware of the relevance of these issues in the shaping of their behaviour and symptoms. Also, they may be unaware of the anomalous experiences they stem from as forms of coping or compensatory reactions. Thus, the value of ocularcentrism as the dominant medium of perception in late modern society contributes to shaping and maintaining the specific form of disembodiment affecting persons with AN. Clinicians, while projecting treatment plans for people affected by this kind of religion we call anorexia, should be aware that the unshakable certainty with which the values of thinness and starvation are held is a shelter in which these persons look for protection from their difficulties in embodiment and in the constitution of their identity in an age dominated by the values of ocularcentrism.

## Lived corporeality and the other: bridging the phenomenological perspective with attachment theory

A serious bias in the scientific literature is the lack of integration between the cognitive–behavioural model and the psychodynamic theories regarding EDs. Even though a plethora of studies focused on the interaction between childhood abuse and neglect with ED psychopathology [27, 102] and its effect on psychological treatments [28, 103, 104], no conclusive models have been proposed to dynamically explain the link between early life experiences and the development of EDs. In this perspective, conceptualising EDs as disorders of embodiment might provide the final keystone between attachment theories and the cognitive-behavioural model. Bruch [105] was one of the first researchers to underline the role of early life experiences in the development of EDs. Ainsworth and Bell [106] showed that the capacity of mothers to respond to the infant’s signals of his needs was related to the quality of the infant's attachment. Attachment theory holds that the repetition of interactions with a caregiver is internalised in the building of affective–cognitive schemas of expectations of care, named internal working models [107, 108]. These models influence the way in which adults experience themselves and others, regulate affects and interact in interpersonal situations; hence, they provide a template for patterns of interpersonal behaviours that are known as adult attachment styles [109]. In this context, the caregiver’s ability to recognise the needs of the infant as differentiated from their own allows the infant to identify their own feelings as separated from the others and to integrate them into a sense of body identity [110]. Indeed, an infant's and young child's relationship with primary caretakers is largely physical, with the mother breastfeeding the baby and the parents bathing, dressing, caressing, kissing, rocking, and cradling the child to nourish, soothe, protect, and respond to the child’s needs, as well as to bond and to express affection toward the child [111]. Thus, first interactive experiences with caregivers, including love, care, responsiveness, and attunement as well as intrusiveness, rejection, maltreatment, and abandonment, are largely communicated through the body during the child’s early years.

According to Bowlby’s attachment theory, nurturing and attuned relational experiences foster positive internal representations of self and others [112, 113]. Furthermore, the interactions between mother and infant in sharing affective states and care provide the first experience and awareness of the body, and provide the original anchor for the development of bodily self-consciousness [114]. Therefore, the body represents the *battleground* and the earliest register for relational experiences. It follows that disturbances in the parent–child relationship, such as intrusive, mis-attuned, unresponsive, or neglectful parenting, abandonment, and prolonged separations, would not only result in insecure attachment, but also interfere with the development of the capacity to embody one’s body and achieve a sense of bodily competence and integrity.

In patients with EDs, the often reported insecure attachment style [115, 116] might be responsible for anomalies in bodily self-consciousness or disordered embodiment, which in turn consist in reduced emotion recognition and ability to feel and decipher bodily “signals”, such as hunger, satiety, fatigue or emotional feelings [85, 87].

**Fig. 3:**
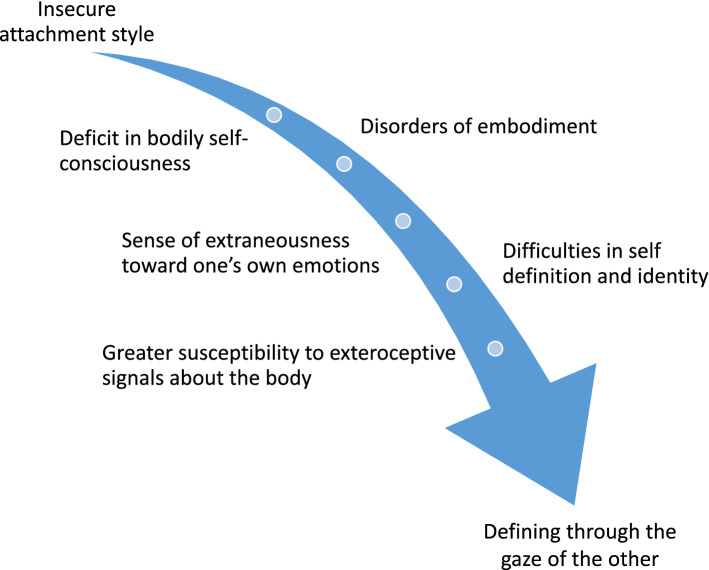
The path from insecure attachment to a disordered embodiment

The step forward in the pathogenetic trajectory is represented by a kind of dysfunctional coping strategy, according to which persons vulnerable to EDs would develop a way to define themselves based on visual representations and on the gaze of the other, rather than on coenaesthesia (Fig. 3). The allocentric lock hypothesis for EDs [117–120] is coherent with the clinical observation that EDs onset is commonly around adolescence [1]. In this crucial age, the self becomes abstracted from the body and is intellectualised as the self-conscious mind [80]. Thus, under physiological conditions, adolescence might result in a matching balance between the egocentric experience and the allocentric one, which is representational and based on abstract knowledge, beliefs, and attitudes related to the body as an object in the third-person perspective [62]. Vulnerability to EDs psychopathology during adolescence might be conceptualised as the development of a predominance of the allocentric representation of the body. This condition is associated with a negative self-image driven by these patients' extreme sensibility to what they experience as the others' disapproving gaze and remarks. Thus, the distorted primary experience of the body is a consequence of the impairment of the process of integration between the egocentric experience of the body and the allocentric representation of it, according to which their body is experienced as an object being looked at by another person [62].

The lived body is no longer a direct, first-personal experiential evidence, but it is an entity that exists as viewed from an external perspective. This means that the other becomes the mirror in which one can perceive oneself [34]. Empirical evidence seems to confirm the relationship between avoidant attachment style with reduced ability to detect bodily signals (including hunger, satiety, fatigue, or emotional feelings), and its interaction with disorders of embodiment, and susceptibility to exteroceptive signals about the body. Monteleone et al. [121] showed that disorders of identity and lived corporeality (IDEA scores) act as a possible mediator between avoidant attachment and specific ED psychopathological traits. The results were confirmed and inserted in a more complex pattern in a further study from the same group, as Cascino et al. [88] demonstrated the centrality of two of the main dimensions of embodiment—feeling extraneous from one’s own body and feeling oneself through objective measures—in a network analysis combining early life experiences, interoceptive awareness and ED specific psychopathology.

Interesting empirical evidence comes from clinical observations of sexuality in patients with EDs [122–124]. Surprisingly, little attention has been devoted to this dimension, even though growing clinical observations demonstrate that the evaluation of sexual functioning might provide information regarding psychopathological features, the recovery process, as well as the etiopathogenetic and pathoplastic trajectory of EDs [125]. More specifically, sexual interactions can be considered as a marker of a person’s capacity to “embody their bodies”, staying connected to and tuned into the body, and experiencing sexual and pleasurable sensations in the body [126]. Sexuality is also “living in and through” the body with the other, getting pleasure from our bodies and experiencing a sense of ownership and agency regarding what is happening during sexual intercourse. Thus, during sexual interactions, persons might also regard themselves as objects to be looked at, inspected, and desired, as a collection of parts meant to be consumed by others—thus assuming the *body-for-the-other* Sartrean perspective. In sexual medicine, *self-objectification* and women’s propensity for self-surveillance has been associated with higher levels of body-image self-consciousness during sexual activity, resulting in dissociation from the immediate moment and detraction from the sexual experience [127]. The resultant self-monitoring of the body’s outward appearance, or self-surveillance [128], fosters increased body shame and appearance anxiety, which trigger negative feelings about the sexual aspects of the self and contribute to sexual dissatisfaction or sexual dysfunction [129]. This phenomenon has been extensively described in patients with EDs and is associated with low sexual desire, with sexuality not lived from within but from a third-person perspective [123]. In a recent study, Cassioli et al. [130] proposed a comprehensive model for the well-known sexual dysfunctions (desire, lubrication and orgasm problems) often detected in patients with EDs and their association with core psychopathology. Authors demonstrated that the interaction between pathological eating behaviours and low sexual desire was explained by disorders of embodiment. This model was in turn mediated by “Discomfort with Closeness”, a pervasive uncomfortable feeling in intimate relationships, a significant factor related to avoidant attachment and associated with fear of intimacy and low incidence of positive experiences in relating with others [131].

To better summarise the empirical results of these studies, it could be useful to report how Lisa (the clinical case mentioned above) described her sexual activity: “We have sex almost every day, and we begin having sex even when I'm not excited. Sometimes, in the middle of the intercourse, I suddenly feel a strange and fearful sensation. I see the scene from above… My body shaking during sex… I perceive a growing feeling of anxiety and pain. At this point I just want him to finish as soon as possible”.

Lisa was an obese girl, neglected by her parents, developing an insecure attachment style. Nobody helped her manage her own emotions, nobody took care of her body, and she learnt that eating was a good way to overcome sadness, loneliness, or anxiety. The gaze of her caregivers (parents, instructors, and even doctors) and the third-person perspective was interiorised: her identity was fixed on body image and weight fluctuation, with regard to high standards of expectations. The loss of a problematic relationship with her boyfriend was managed in the only way she could afford: dieting and controlling her weight. In this prototypical case, we can understand that an ED is like a carsic river: the disorder of embodiment is like underground flowing water, emerging one time as obesity, another as AN, BN or as a disorder of sexuality. Recovery from an ED means achieving a healthier relationship with one’s own body, which implies the recovery of a renewed capacity to create intimate bondages and to experience one’s own body without frightening.

## Implications for treatment

According to the National Institute for Health and Care Excellence (NICE), Cognitive Behavioural Therapy-Enhanced (CBT-E) is recommended as the first-line treatment in managing patients with AN, BN and BED [132]. Other psychological interventions, such as Interpersonal psychotherapy (IPT), Family therapy and family interventions and Focal Psychodynamic therapy, are also suggested, and several authors supported the equivalent efficacy of these interventions as compared with CBT-E [133, 134]. Based on available evidence, none of the third-wave therapies such as Schema Therapy met established criteria for an empirically supported treatment for particular ED subgroups [135]. Since its first development, Fairburn’s trans-diagnostic model [136] of maintenance of EDs has been widely adopted as the theoretical frame for psychological interventions aimed at interrupting pathological eating behaviours. The treatment protocol based on Fairburn’s model resulted to be very effective in reducing symptoms such as binge/purging or severe dieting in the short–midterm [137]. However, despite the clear evidence of improved efficacy of the new version of CBT, the CBT-E [138, 139], several largely shared clinical observations should be considered: there is an extraordinary heterogeneity of response rate, depending on the duration of illness, concurrent psychiatric disorders, history of abuse and other potential moderators of treatment [140]. Furthermore, the long-term remission rate is less than 50% [141, 142], partly due to the high relapse rate [2]. Moreover, long-term observations challenged the actual definition of what recovery from an EDs actually is [15]: undoubtedly, CBT-E is efficacious on a behavioural level. However, it is possible that the high relapse and chronicity rate can be attributed to the less clear efficacy on a more profound psychopathological core. Very few empirical studies reported evidence regarding the mediators of the efficacy of treatment interventions on EDs psychopathology [143, 144]. CBT-E is first and foremost aimed at interrupting abnormal behaviours, such as starvation, binge-eating and body checking, through the disputing of the distorted beliefs related to body shape and weight. Therefore, it seems that the research is also biased on an optical-coenaesthetic disproportion in its choice of treatment target and treatment mediators [145]. Even though new targets of interventions have been added in CBT-E as compared to previous models (e.g. specific intervention of emotion dysregulation), available treatments do not include specific embodiment-focused modules [146].

In an attempt to overcome this limitation, Rossi et al. demonstrated that after a multidisciplinary treatment including CBT-E, the variation of the embodiment disorder represented a possible mediator of the efficacy of CBT-E on ED symptoms in AN [146]. In other words, the longitudinal mediation model showed that patients reporting a stable reduction across time of behavioural symptoms as well body uneasiness were those who showed a significant reduction of the embodiment disorder, confirming previous empirical observations on the pivotal role of the embodiment disorder in ED psychopathology [34, 82, 88, 90]. Authors hypothesised that the reason why the amelioration of the embodiment disorder determined a reduction of the overvaluation of body shape/weight and of body uneasiness might be the fact that the renewed confidence in the stability of their own body in space and time and the restoration of their identity might help patients to start looking again beyond their own corporeality, widening their horizon of values [146, 147].

New issues for research are raised by these findings: for example, whether the recovery of a healthier lived corporeality is due to the so-called non-specific therapeutic factors [148] or, alternatively, linked to specific effects determined by the interruption of abnormal behaviours, such as starvation, binge-eating and body checking, and by the disputing of the distorted beliefs related to feeding and body shape and weight. Even though preliminary, the results of this study suggest that new treatments which integrate CBT-E with the phenomenological concepts related to the optical-coenaesthesic disproportion hypothesis might improve the recovery rate of EDs in the long term.

First, the assessment of patients with EDs should include an evaluation of experiential features, overcoming the mere evaluation of pathological eating behaviours [149]. In particular, the apprehension of one’s own body should be investigated taking into account all three aforementioned dimensions of corporeality: subject-body, object-body, and body-for-others. The difficulties in recognising inner body signals and emotions should be noted, as well as the extent of the role of objective measures in determining a sense of stability of one’s own body in space and time. Moreover, the impact of the gaze of the others as well as of the illness on the definition of one’s own identity should not be underestimated. A phenomenological assessment should also include the patient’s perception of space, of the body in space, and of time, especially associated with pathological eating behaviours, including binge-eating and body checking. Integrative therapeutic modules should be focused on these dimensions of corporeality. In particular, interoceptive deficits of patients with EDs should be specifically targeted [150]. Indeed, the recovery of a healthier relationship with inner bodily signals could represent the first step in the complicated process of recovering a healthier contact with emotions [151] and thus with one’s own desires and values, allowing the patients to overcome the definition of one’s own Self as an “Anorexic Person” [62]. In this field, new therapeutic interventions such as Mindful Awareness in Body-Oriented Therapy (MABT) [152] and PHD psychotherapy [149] might be promising. Moreover, the association of top–down cognitive techniques aimed at deepening the exploration of the dimensions of Selfhood and Identity might be helpful [153], extending the work on the domains of self-evaluation proposed by Fairburn’s treatment model. Furthermore, considering the crucial role of adverse childhood experiences and of insecure attachment style in the development of the embodiment disorder in patients with EDs [68, 89], it could be useful to integrate modules focused on evolutionary aspects. An integrated psychotherapeutic approach targeting all these features might make it possible to overcome the limitations of the existing treatments, improving the prognosis of these patients by taking into account in a single multidimensional psychopathological and treatment model the cognitive-ideational dimensions of ED psychopathology as well as the emotional, perceptual, experiential, relational and identity-related issues that all together constitute the roots and flowering branches of these severe disorders.

## Strengths and limitations

The present review article represents one of the few attempts of combining cognitive, phenomenological, and psychodynamic approaches to eating disorders, opening tracks for future research. The article provides an interpretation of pathological eating behaviours according to different perspectives, taking into account the impact of philosophy. While the importance of the embodiment disturbance in the clinical practice is well documented, the article highlights the possible enrichment caused by phenomenological and psychodynamic approaches to EDs psychopathology, by means of the empirical evidence provided by the IDEA questionnaire. New implications for treatment come out of this more differentiated and complex approach of EDs psychopathology.

The major limitations are the shortage of empirical (especially prospective) studies regarding the IDEA questionnaire, and the biological correlates of the proposed model. Several facets of the conceptual model are still in speculation phases. It should also be noted that most of the scientific evidence on this subject relates to females, as the prevalence of EDs in males is much lower. Therefore, some of the arguments reported and discussed may not be generalizable to male subjects with EDs. Finally, considering the importance of traumatic experiences in the pathogenesis of EDs, the complex interplay between childhood trauma and embodiment disorder is still far to be elucidated.

## What is already known on this subject?

The cognitive–behavioural model is the most studied and, up to now, clinically efficacious treatment for EDs. However, as any coherent and scientifically grounded model, it presents some limitations in its application. Numerous patients report a chronic course, do not respond to treatment and develop a personality structure based on pathological eating behaviours, since “being anorexic” becomes a new identity for the person. Furthermore, the etiopathogenetic trajectory of EDs influences the treatment response: for example, patients reporting childhood abuse or maltreatment respond differently to cognitive–behavioural therapy.

## What does this study add?

To obtain a deeper comprehension of these disorders, it seems important to shift attention from abnormal eating behaviours to more complex and subtle psycho(patho)logical features, especially experiential ones. Thus, the present review aims to provide an integrated view of cognitive, psychodynamic, and phenomenological perspectives on EDs, suggesting new therapeutic targets and intervention strategies based on this integrated model.

## Data Availability

Not applicable.
